# Case Report: Paroxysmal autonomic instability with dystonia syndrome: a rare complication of tuberculous meningitis

**DOI:** 10.12688/f1000research.141196.1

**Published:** 2023-10-25

**Authors:** Lowrence Precious Dichoso, Gerald Pagaling, Roland Dominic G. Jamora, Veeda Michelle M. Anlacan

**Affiliations:** 1 Department of Neurosciences, College of Medicine, Philippine General Hospital, University of the Philippines, Manila, NCR, 1004, Philippines; 2 Institute for Neurosciences, St Lukes Medical Center, Bonifacio Global City, Taguig, NCR, 1635, Philippines; 3 3Department of Neurology, Institute of Neurological Sciences, The Medical City, Pasig City, NCR, 1600, Philippines

**Keywords:** Paroxysmal autonomic instability with dystonia case report; Tuberculous meningitis; Dystonia; PAIDS

## Abstract

Paroxysmal autonomic instability with dystonia syndrome (PAIDS) is a rare and life-threatening complication of neurologic diseases. We report the case of a 20-year-old male with acute severe brain damage from tuberculous meningitis, who eventually developed paroxysmal episodes of spontaneous and inducible tachycardia, tachypnea, hypertension, and decerebrate posturing. We diagnosed the patient as suffering from paroxysmal autonomic instability with dystonia syndrome. The unavailability of morphine and the prohibitive cost of prolonged fentanyl use led to a trial of gabapentin, clonazepam, and propranolol for the patient, resulting in symptom resolution. Brain injury causes dysfunction of autonomic centers leading to paroxysmal autonomic instability with dystonia syndrome. Management includes minimizing stimulation and pharmacotherapy with preventive and abortive medications. Alternatives like gabapentin, propranolol and clonazepam were effective in treating the paroxysmal episodes, proving that they may have a role in resource limited settings. PAIDS requires urgent recognition and treatment to prevent further complications and death.

## Introduction

Paroxysmal autonomic instability with dystonia syndrome (PAIDS) is a relatively uncommon complication of various central nervous system diseases presenting as a constellation of episodic autonomic hyperactivity accompanied by dystonia, occurring at least one cycle per day for more than three days. ^1^^–^^3^ The autonomic dysfunction is caused by disruption of diencephalic connections causing disinhibition or increased activation of the lower sympathetic activating center. ^1^^,^^4^^,^^5^ The dystonic posturing is from a midbrain lesion causing disconnection of the normal inhibitory signals to the pontine and vestibular nuclei. ^4^ We report a patient with PAIDS secondary to tuberculous meningitis, successfully managed with gabapentin, clonazepam, and propranolol.

## Case report

**Figure 1.**
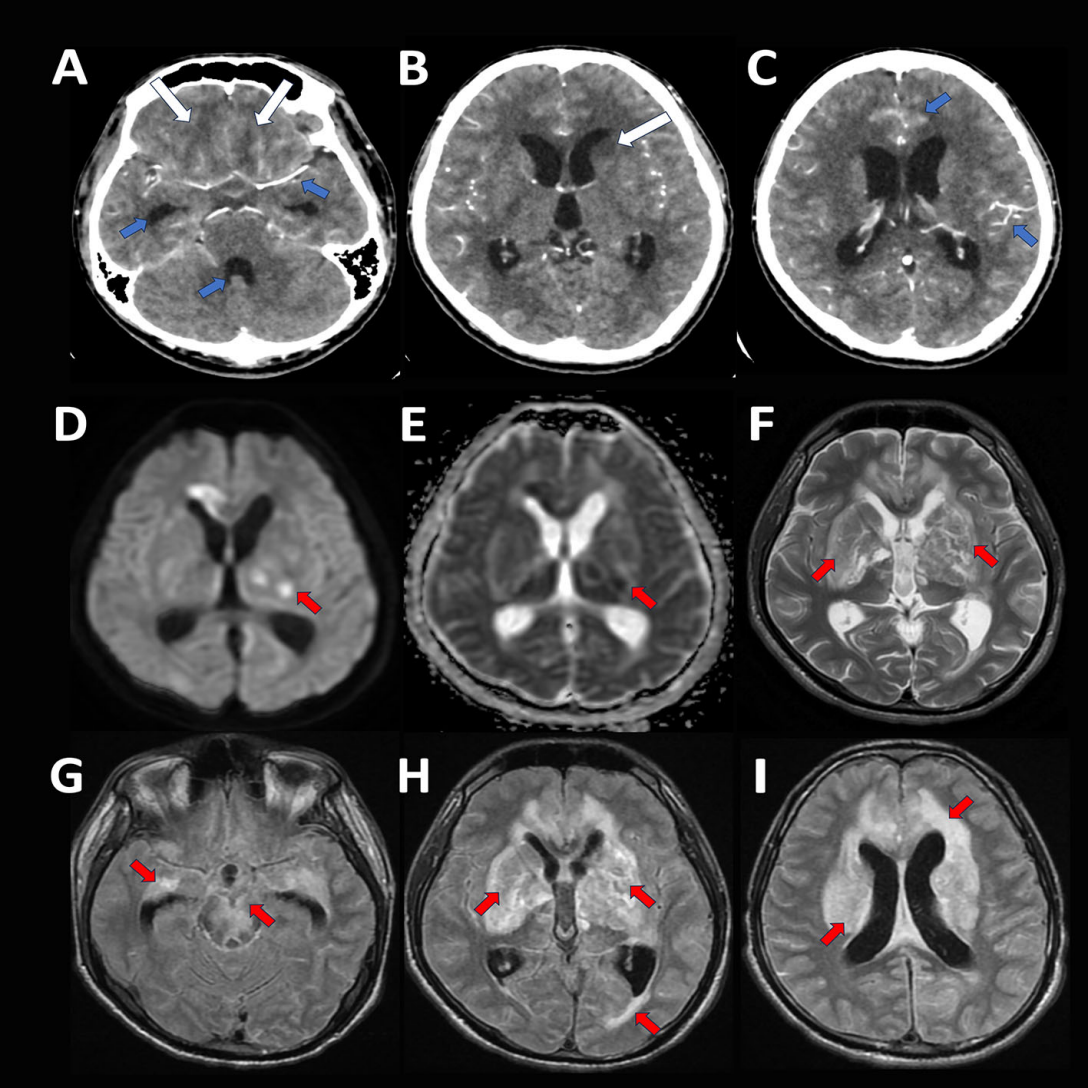
Neuroimaging of the patient. Cranial computed tomography scan with contrast (A-C): Fairly defined focal areas of hypodensities seen in the deep white matter of the bilateral basofrontal parasagittal lobes, head of the left caudate nucleus, and anterior limb of the left internal capsule (A,B: white arrows), with undue leptomeningeal enhancement and mild communicating hydrocephalus (A,C: blue arrows). Cranial MRI with contrast (D-I): Hyperintense foci with some areas of restricted diffusion and magnetic susceptibility artifacts with surrounding vasogenic edema in the cortical-subcortical junction of the bilateral basal ganglia, thalamus, midbrain and pons, bilateral temporal, bilateral parietal, left occipital lobes and bilateral frontal lobes (D-I: red arrows); with leptomeningeal and pachymeningeal nodular thickening and enhancement.

A 20-year-old Filipino male student without comorbidities was admitted to our institution for a three-week history of behavioral changes accompanied by incoherent speech, waddling gait, febrile episodes, anorexia, and generalized weakness. He had two episodes of generalized tonic seizures lysed by diazepam at the emergency room. On examination, he had no eye-opening to pain, non-purposeful movements of all extremities, bilateral extensor toe sign and a supple neck. Chest X-ray revealed reticular opacities on both upper lung fields. A cranial computed tomography (CT) scan with contrast was suggestive of tuberculous meningitis with cerebritis (see Figure 1A-C). The lumbar tap revealed a clear yellow cerebrospinal fluid (CSF) with elevated opening pressure, pleocytosis of 8×10 ^6^/L (neutrophils 20%, lymphocytes 60%), elevated protein of 687 mg/dL, hypoglycorrhachia of 1% of serum, and a negative Cryptococcal Antigen Latex Agglutination System, Bactigen Latex Agglutination, and fungal culture. He was diagnosed with disseminated tuberculosis with brain and lung involvement and was started on isoniazid/rifampicin/pyrazinamide/ethambutol tablets plus dexamethasone. A therapeutic lumbar tap was done every 2-3 days in the absence of consent for a ventriculoperitoneal shunt (VPS) placement.

**Figure 2.**
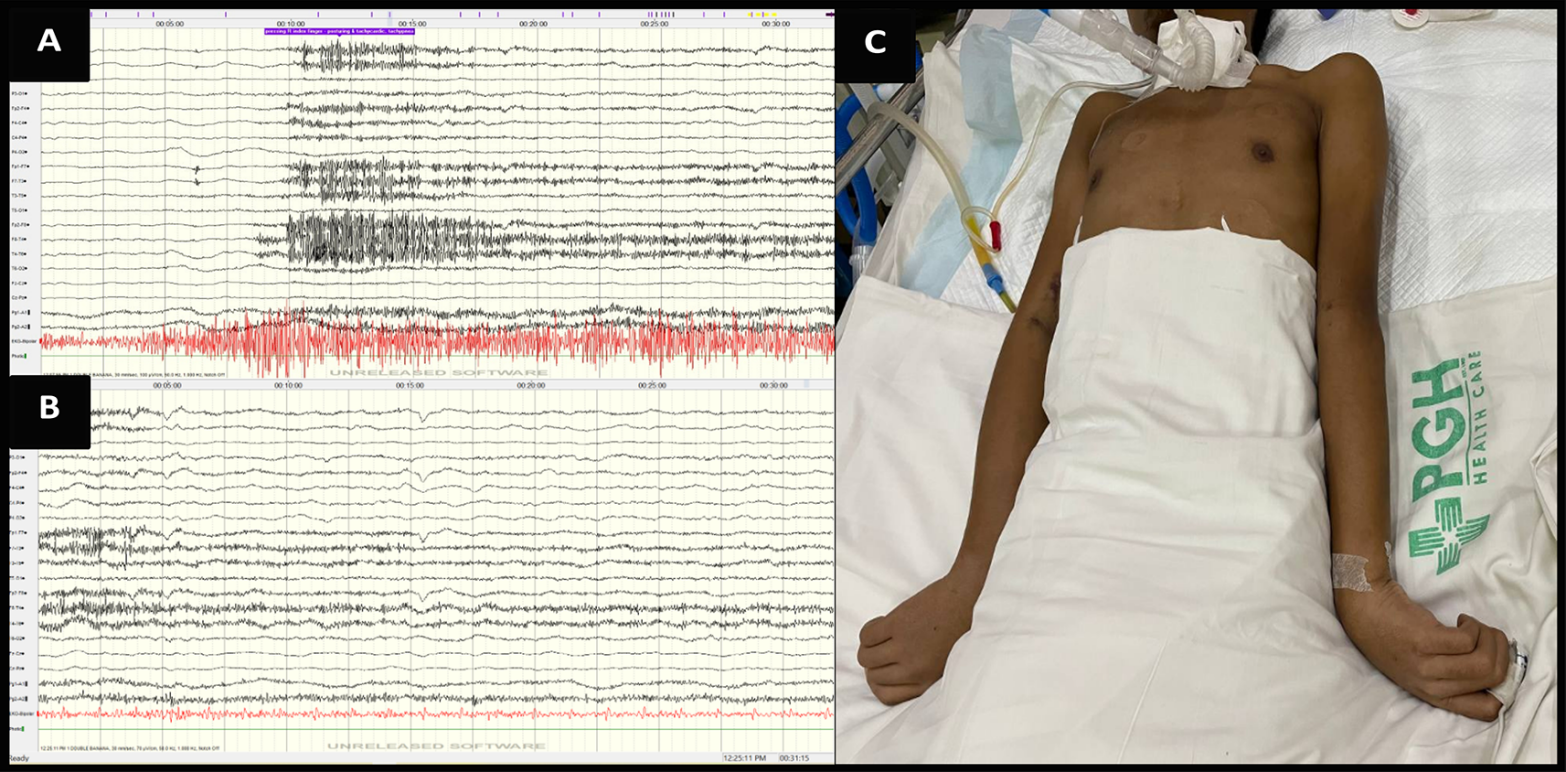
21-channel electroencephalogram. A, B: Epoch of electroencephalogram at the start during (A), and after the paroxysmal episodes showing absence of epileptiform discharges (B) prior to the event as evidenced by muscle artifacts. C: The decerebrate posturing of the patient during the event.

On the tenth hospital day, the patient developed spontaneous and inducible paroxysmal episodes of tachycardia (>130 beats/minute), tachypnea (>40 breaths/minute), hypertension (150/90 mmHg) with associated extensor posturing of both upper extremities lasting for 1-3 minutes and occurring 3-5 times a day. These were noted during patient manipulations like suctioning and bed turning. The Paroxysmal Sympathetic Hyperactivity Assessment Measure (PSH-AM) was used. This is composed of an 11-point Diagnosis Likelihood Tool (DLT) and a Clinical Feature Scale (CFS) to guide the diagnosis, which has a corresponding likelihood and severity score, respectively. ^1^ With this criterion, our case was very likely to be PAID syndrome with a total score of more than 17 (DLT = 8 points + CFS = 13 points, with a final PSH-AM score of 21). ^4^ Cranial magnetic resonance imaging with contrast revealed a further extension of the tuberculous abscess and non-resolution of hydrocephalus (see Figure 1D-I). A 21-channel electroencephalogram revealed diffuse slowing of background activity, absence of epileptiform discharges with no electrographic correlate of the extensor posturing of both upper extremities, likely dystonia (see Figure 2A-C). Fentanyl drip was started due to unavailability of morphine. However, this was eventually shifted to gabapentin 600 mg three times a day and propranolol 30 mg four times a day. The uptitration of propranolol was limited by episodes of hypotension hence clonazepam 2 mg twice a day was added. The frequency and duration of the paroxysms was reduced to 1-2 episodes per day. By the fifth week of treatment, there was complete resolution of all symptoms. The patient was discharged on a nasogastric tube and on room air via a tracheostomy, with a modified Rankin Score (mRS) of 5 and Barthel Index of Activities of Daily Living score of 1/20. On follow-up via telemedicine after 3 months, the patient has spontaneous purposeful movements of all extremities. He was still maintained on gabapentin, while propranolol and clonazepam were tapered off, without recurrence of autonomic instability and dystonia. The mRS and Barthel Index score remained the same. The patient’s family was able to continue with the treatment due to its lower cost.

## Discussion

PAIDS is a syndrome of sympathetic hyperactivity accompanied by abnormal posturing. ^6^ It is commonly observed after a severe traumatic brain injury (TBI) (79.4%); occurring in almost one-third of all cases of TBI but may also arise from paraneoplastic limbic encephalopathy (9.7%), cerebrovascular disorders (5.4%), hydrocephalus (2.6%), tumor (0.6%), and rarely after central nervous system infections (0.3%) like in pneumococcal meningoencephalitis and tuberculous meningitis, as in our case. ^2^^,^^4^^,^^7^

The pathophysiology of PAIDS is still not well understood but likely best explained by the Disconnection Theory, wherein a brain injury causes dysfunction of autonomic centers in the diencephalon. ^5^ Due to the disconnection of higher sympathetic inhibitory regions ( *e.g.* insula, cingulate cortex) to the lower sympathetic activating centers (hypothalamus to the lower brainstem), there is increased activation of the excitatory pathways, triggering catecholamine release, and the subsequent hyperadrenergic state. ^4^^,^^5^^,^^8^ It could occur from damage to the descending inhibitory pathways coming from the forebrain and rostral brainstem leading to a lack of inhibition that releases spinal sympathoexcitatory reflexes that then overreact to peripheral stimulation, explaining the paroxysms seen mostly during stimulation. ^4^ Lesions in the midbrain that cause blockage of normal inhibitory signals to the pontine and vestibular nuclei result in rigidity and decerebrate posturing. ^2^^,^^9^^,^^10^ Other than the patient’s tuberculous meningitis, the concomitant hydrocephalus could also have contributed to the development of the PAIDS. Even if a VPS was not done, there was no increase in the size of the hydrocephalus after repeat imaging, therefore, dysfunction of diencephalic and upper midbrain circuits and impaired inhibition of medullary sympathoexcitatory neurons from brainstem ischemia are thought to be the cause of the patient’s symptoms. ^4^ On review of literature, PAIDS was reported in a child with tuberculous meningitis with note of persistence of symptoms despite the placement of a VPS; suggesting that the tubercular infiltration in the autonomic centers was the inciting cause. ^10^

PAIDS is a life-threatening condition that may lead to secondary hypertensive or hyperthermic encephalopathy and/or death. Differentiation from a neuroleptic malignant syndrome, malignant hyperthermia, sepsis, seizures, and impending herniation is very important. ^6^ In our case, the diagnosis of PAIDS was made using the PSH-AM. ^4^

Management includes minimizing stimulation and pharmacotherapy with preventive and abortive medications. The available data on treatment options are lacking. The clinical symptoms and treatments of PAID from tuberculous meningitis are presented in Table 1. Morphine sulfate was found to be most effective in preventing paroxysms, and other proposed medications include baclofen, benzodiazepines, bromocriptine, clonidine, dexmedetomidine, gabapentin, and propranolol. Data are mostly from small case series or case reports of PAID syndrome from TBI. ^4^

**Table 1.** Reports of paroxysmal autonomic instability with dystonia syndrome (PAIDS) in patients with tuberculous meningitis.

Authors, Year	Age/Sex	Onset of PAIDS from tuberculous meningitis	Signs and symptoms	Treatment	Outcome
Ramdhani *et al.* ^2^ (2010)	69/M	More than 9 ^th^ day from admission	Hypertension, tachycardia, tachypnea, transpiration, desaturations, high fever, pink foamy sputum, and dystonia lasting for 30 minutes	**Abortive:** morphine benzodiazepines clonidine **Preventive:** β-blocker clonidine	• Cessation of paroxysms 1 month after admission • Glasgow coma score (GCS) of E4V4M5 but neurologically severely impaired • Died after three months from pneumonia
Sarkar *et al.* ^3^ (2011)	3/F	4 ^th^ day of admission	Hypertension, tachycardia, diaphoresis, tachypnea, hyperthermia, and dystonia with extensor posturing lasting for 30 minutes	**Abortive:** benzodiazepine **Preventive:** propranolol	• Cessation after weeks of treatment • GCS of E4V4M5 however remained neurologically impaired on follow-up
Singh *et al.* ^10^ (2012)	1/F	More than 3 days from admission	Tachycardia, paroxysms of tachypnea, diaphoresis, and extensor posturing lasting for 30 minutes	**Abortive:** benzodiazepine **Preventive:** β-blocker clonidine	• Paroxysms ceased after 1 month from admission • Improvement of neurologic status and was neurologically stable after 3 months of follow-up

Persistent and severe episodes of sympathetic hyperactivity can cause catastrophic consequences such as worsening of intracranial hypertension or hemorrhage, posterior reversible encephalopathy syndrome, stress-induced cardiomyopathy, hypoxic respiratory failure, acute tubular necrosis, renal failure, and rhabdomyolysis. ^4^ Pharmacologic treatment can also cause iatrogenic autonomic dysfunction leading to an exaggerated cardiac and vascular response. PAIDS has poorer clinical outcomes and increased morbidity when associated with generalized motor rigidity and poor cognitive function. ^1^^,^^4^^,^^5^ There is only one case report of PAIDS from tuberculous meningitis in an adult, hence there is limited knowledge on the expected outcome and subsequent quality of life after recovery. ^2^

## Conclusions

PAID is a rare complication of tuberculous meningitis that can mimic other life-threatening conditions, hence early detection is vital to avoid unnecessary diagnostics and treatments. The diagnosis is challenging and exacting in a resource-limited country and early recognition is crucial for immediate, adequate, and cost-effective treatment.

### Patient consent

Written informed consent was obtained from the patient’s authorized representative (mother) for the publication of this case report and any accompanying images.

The research was conducted ethically in accordance with the World Medical Association Declaration of Helsinki.

## Data Availability

No data associated with this article.
