# Supplementary material for: Case Report: Paroxysmal autonomic instability with dystonia syndrome: a rare complication of tuberculous meningitis
Source: F1000Res. 2023 Oct 25;12:1405. [Version 1] doi: 10.12688/f1000research.141196.1 (PMC11377918; doi:10.12688/f1000research.141196.1)
Supplement: Peer review history

# Reviewer report: Reviewer response for version 1

**DOI:** 10.5256/f1000research.154617.r274520

**Published:** 2024-09-05

**Authors:** Nor Osman Sidow

**Affiliations:** 1 Mogadishu Somalia Turkey Training and Research Hospital, Mogadishu, Somalia

This case is good and it explains in details of the information provided.

 the background and discussion is quite is enough  and I  agree that it is suitable for indexing

 the language is excellent 

 the case also is novel and perfect 

 the quality of the images and figures are very good

Are enough details provided of any physical examination and diagnostic tests, treatment given and outcomes?

Yes

Is the case presented with sufficient detail to be useful for other practitioners?

Yes

Is sufficient discussion included of the importance of the findings and their relevance to future understanding of disease processes, diagnosis or treatment?

Yes

Is the background of the case’s history and progression described in sufficient detail?

Yes

Reviewer Expertise:

Neurology and Clinical Neurophysiology

I confirm that I have read this submission and believe that I have an appropriate level of expertise to confirm that it is of an acceptable scientific standard.

# Reviewer report: Reviewer response for version 1

**DOI:** 10.5256/f1000research.154617.r274512

**Published:** 2024-06-04

**Authors:** Shailendra Katwal

**Affiliations:** 1 National Trauma Center, Kathmandu, Nepal; 2 Dadeldhura Subregional Hospital,, Dadeldhura,, Nepal

In neuroimaging figures, captions doesn’t explain the findings correctly. Not all the sequences are DWI/ADC. Mention the sequences and findings correctly. Even the right sided blue arrows in figure A are showing the ventricles instead of meningeal enhancement.

 Findings of reticular opacity in lung doesn’t mean the active form of pulmonary tuberculosis, however its mentioned as disseminated tuberculosis with brain and lung findings.

  What are the differential of this clinical entity and how do your rule out them?

Are enough details provided of any physical examination and diagnostic tests, treatment given and outcomes?

No

Is the case presented with sufficient detail to be useful for other practitioners?

Yes

Is sufficient discussion included of the importance of the findings and their relevance to future understanding of disease processes, diagnosis or treatment?

Yes

Is the background of the case’s history and progression described in sufficient detail?

Yes

Reviewer Expertise:

NA

I confirm that I have read this submission and believe that I have an appropriate level of expertise to confirm that it is of an acceptable scientific standard, however I have significant reservations, as outlined above.

# Reviewer report: Reviewer response for version 1

**DOI:** 10.5256/f1000research.154617.r269320

**Published:** 2024-05-09

**Authors:** Venkat Ramesh

**Affiliations:** 1 Apollo Hospitals, Hyderabad, Telangana, India

Overall, the case report has been satisfactorily written, and provides a novel insight.

 The exact CSF glucose value needs to be mentioned.

 How the diagnosis of CNS TB was established needs to be elaborated in detail. . Was a GeneXpert M.tb (or other CB-NAAT) done? Was a bronchoscopy performed? 

 The case report needs minor modifications.

Are enough details provided of any physical examination and diagnostic tests, treatment given and outcomes?

Partly

Is the case presented with sufficient detail to be useful for other practitioners?

Yes

Is sufficient discussion included of the importance of the findings and their relevance to future understanding of disease processes, diagnosis or treatment?

Yes

Is the background of the case’s history and progression described in sufficient detail?

Yes

Reviewer Expertise:

Tuberculous meningitis

I confirm that I have read this submission and believe that I have an appropriate level of expertise to confirm that it is of an acceptable scientific standard, however I have significant reservations, as outlined above.
